# Supplementary material for: Mechanisms governing the pioneering and redistribution capabilities of the non-classical pioneer PU.1
Source: Nat Commun. 2020 Jan 21;11:402. doi: 10.1038/s41467-019-13960-2 (PMC6972792; doi:10.1038/s41467-019-13960-2)
Supplement: Supplementary file 7 — Source data [file 41467_2019_13960_MOESM7_ESM.zip › Source_Data/Figure6/Figure6B_MotifScanOutput/ETS1_PU.1/homerResults/motif1.similar.html]

motif1

## Information for motif1

C
T
G
A
C
T
G
A
T
A
G
C
T
G
C
A
C
T
A
G
A
C
T
G
C
G
T
A
C
G
T
A
T
C
A
G
G
A
C
T
T
C
A
G
  
Reverse Opposite:  

A
G
T
C
C
T
G
A
A
G
T
C
G
C
A
T
A
C
G
T
A
G
T
C
A
G
T
C
A
C
G
T
A
T
C
G
G
A
C
T
G
A
C
T
  

|  |  |
| --- | --- |
| p-value: | 1e-3181 |
| log p-value: | -7.325e+03 |
| Information Content per bp: | 1.587 |
| Number of Target Sequences with motif | 12519.0 |
| Percentage of Target Sequences with motif | 79.08% |
| Number of Background Sequences with motif | 7123.7 |
| Percentage of Background Sequences with motif | 22.21% |
| Average Position of motif in Targets | 101.1 +/- 44.9bp |
| Average Position of motif in Background | 99.9 +/- 71.9bp |
| Strand Bias (log2 ratio + to - strand density) | 0.0 |
| Multiplicity (# of sites on avg that occur together) | 1.67 |
| Motif File: | file (matrix) reverse opposite |

### Similar de novo motifs found

|  |  |  |  |  |  |  |  |
| --- | --- | --- | --- | --- | --- | --- | --- |
| Rank | Match Score | Redundant Motif | P-value | log P-value | % of Targets | % of Background | Motif file |
| 1 | 0.981 | C T G A A G T C C G A T A C G T A G T C A G T C A C G T A T C G G A C T G A C T | 1e-3067 | -7062.588963 | 75.81% | 20.16% | motif file (matrix) |
| 2 | 0.944 | T A G C T G A C A C T G A C T G C G T A C G T A T C A G G A C T T C A G T C A G | 1e-2874 | -6617.982667 | 92.16% | 40.68% | motif file (matrix) |
| 3 | 0.959 | T G A C T G C A C T A G A C T G C G T A G C T A T C A G G A C T | 1e-2866 | -6599.632446 | 84.47% | 30.58% | motif file (matrix) |
| 4 | 0.964 | C T G A A G T C C G A T A C G T A G T C A G T C A C G T A T G C G A C T | 1e-2798 | -6443.914689 | 74.30% | 21.02% | motif file (matrix) |
| 5 | 0.988 | A G T C A G T C C T G A A G T C C G A T A C G T A G T C A G T C A G C T A T G C G A C T A G C T | 1e-2721 | -6266.592728 | 80.32% | 27.42% | motif file (matrix) |
| 6 | 0.920 | C T G A A G T C C G A T A C G T A G T C A G T C A G C T | 1e-2548 | -5867.957738 | 74.23% | 23.13% | motif file (matrix) |
| 7 | 0.957 | A G T C G A T C C T G A A G T C C G A T C G A T A T G C A G T C A C G T | 1e-2315 | -5332.362565 | 95.05% | 51.90% | motif file (matrix) |
| 8 | 0.921 | A T G C G T A C C T A G A C T G G T C A C G T A C T A G A G T C T A G C A T C G T A G C A T G C | 1e-2127 | -4898.554853 | 68.52% | 22.02% | motif file (matrix) |
| 9 | 0.900 | T A G C C T A G A T C G G C T A C G T A T C A G A G C T C A T G | 1e-1921 | -4424.660457 | 90.15% | 48.59% | motif file (matrix) |
| 10 | 0.877 | C T A G T G A C C T G A A C T G A C T G C G T A C G T A T G A C A G C T C A T G T G C A T C G A C T G A T G C A | 1e-1907 | -4391.848962 | 68.74% | 24.42% | motif file (matrix) |
| 11 | 0.891 | A G T C C A G T A C G T A G T C T A G C A C T G A T C G G A C T | 1e-1856 | -4274.004468 | 54.57% | 13.88% | motif file (matrix) |
| 12 | 0.869 | A G T C G A T C G C T A A G T C G C A T C G A T A G T C A G T C A G C T A T G C G A C T A G C T G A T C | 1e-1693 | -3899.222180 | 61.48% | 20.52% | motif file (matrix) |
| 13 | 0.858 | A G T C C G A T C G A T A T G C A G T C A C T G A T C G A G T C A T G C | 1e-1621 | -3734.193253 | 57.08% | 17.84% | motif file (matrix) |
| 14 | 0.916 | C T G A T A G C G T C A A C T G A C T G C G T A C G T A C T A G A G T C G T A C C T G A T G A C C G T A | 1e-1522 | -3505.656190 | 55.22% | 17.45% | motif file (matrix) |
| 15 | 0.883 | A G T C G A T C G C T A A G T C G C A T A C G T A G T C G A T C A G C T A T G C G A C T A G C T A G T C G A T C | 1e-1510 | -3478.603349 | 64.42% | 25.02% | motif file (matrix) |
| 16 | 0.847 | C G T A T C G A A T C G A C G T A C G T A G T C A G T C A G C T A T C G A C G T A C T G G C T A T C G A | 1e-1477 | -3402.163031 | 58.40% | 20.36% | motif file (matrix) |
| 17 | 0.896 | G T A C T A G C A G C T A G T C C T G A A G T C A C G T A C G T A G T C A G T C A G C T A T C G T G A C | 1e-1421 | -3274.224777 | 39.80% | 8.31% | motif file (matrix) |
| 18 | 0.888 | A T G C A C G T A C G T A G T C A G T C A G C T A T G C | 1e-1285 | -2959.124897 | 74.62% | 37.89% | motif file (matrix) |
| 19 | 0.889 | G A C T C A G T A T G C A G T C A C G T A T G C G A C T A G C T | 1e-1230 | -2833.989132 | 67.25% | 31.21% | motif file (matrix) |
| 20 | 0.890 | C T G A A G C T A C G T A G C T A G T C A T G C A T C G | 1e-1009 | -2323.817717 | 63.29% | 30.69% | motif file (matrix) |
| 21 | 0.816 | T G A C G T A C C G T A A G T C C G T A A C G T A G T C A G T C A G C T T C A G G A T C | 1e-1006 | -2317.067154 | 24.63% | 3.66% | motif file (matrix) |
| 22 | 0.842 | A T G C C G A T A C G T A G T C A G T C A G T C G A T C G C A T A C G T A G C T | 1e-905 | -2085.794739 | 47.18% | 18.54% | motif file (matrix) |
| 23 | 0.825 | A T C G C G A T A C G T T A G C A G T C A C T G A G T C C G A T G C A T G C T A | 1e-892 | -2054.095238 | 42.91% | 15.54% | motif file (matrix) |
| 24 | 0.836 | C T G A A T G C G A C T A C G T A G T C A G T C A G T C G A T C G A C T G A C T A G C T A G C T | 1e-880 | -2028.445359 | 42.66% | 15.50% | motif file (matrix) |
| 25 | 0.838 | A T G C A C G T C G A T A G T C A G T C A T C G A G T C C G T A G C A T | 1e-752 | -1733.746157 | 38.85% | 14.43% | motif file (matrix) |
| 26 | 0.830 | A G C T A G C T A T G C A C G T A G C T A G T C G A T C A C G T A T G C G A C T A C G T A G C T A T G C | 1e-582 | -1340.934734 | 27.05% | 8.68% | motif file (matrix) |
| 27 | 0.822 | T G A C C G T A A T G C G C A T C G A T A T G C G A T C G C A T G A T C G T A C | 1e-574 | -1322.634692 | 21.98% | 5.75% | motif file (matrix) |
| 28 | 0.801 | G T C A C G T A A T C G T G C A C T A G A C T G G C T A C T G A A T C G | 1e-478 | -1102.261864 | 24.95% | 8.64% | motif file (matrix) |
| 29 | 0.819 | T C G A A T G C C A G T A C G T A G T C G A T C T A C G A G T C G C A T | 1e-409 | -943.056341 | 25.74% | 10.15% | motif file (matrix) |
| 30 | 0.774 | G C A T C G A T A G T C A G T C A T C G A T G C G C A T G A C T A G C T | 1e-407 | -937.298794 | 18.77% | 5.72% | motif file (matrix) |
| 31 | 0.859 | C G T A A T G C A G C T A C G T A G T C G T A C A G C T C G T A G A C T G C A T G C A T | 1e-398 | -916.734056 | 15.40% | 3.92% | motif file (matrix) |
| 32 | 0.829 | G C T A T C G A A T G C C G A T A C G T T G A C A G T C A G C T C G T A G A C T | 1e-395 | -910.897737 | 15.16% | 3.81% | motif file (matrix) |
| 33 | 0.816 | C A G T T C A G T C A G T G A C A G T C C A T G A T C G T C G A C G T A T C A G A G C T A G T C C A T G T A G C | 1e-268 | -618.304074 | 10.94% | 2.86% | motif file (matrix) |
| 34 | 0.811 | C A G T C T A G A C T G A G C T A C G T A G T C A G T C A G C T A T G C A G C T A G C T A C G T | 1e-148 | -341.933867 | 8.55% | 2.95% | motif file (matrix) |
| 35 | 0.768 | C G T A A C T G A C G T A C G T A G T C A G T C A G C T | 1e-131 | -303.320157 | 14.79% | 7.50% | motif file (matrix) |
| 36 | 0.771 | C T G A A T G C C G T A A C T G A C T G C G T A C G T A C T A G A C T G C T G A | 1e-95 | -219.406066 | 3.75% | 0.91% | motif file (matrix) |
| 37 | 0.800 | G A T C T C G A A G T C G A C T C A G T T A G C G T A C A G C T C G T A G A C T G A C T G A C T | 1e-72 | -166.387680 | 3.59% | 1.09% | motif file (matrix) |
